# Combination of Cannabinoids, Δ9- Tetrahydrocannabinol and Cannabidiol, Ameliorates Experimental Multiple Sclerosis by Suppressing Neuroinflammation Through Regulation of miRNA-Mediated Signaling Pathways

**DOI:** 10.3389/fimmu.2019.01921

**Published:** 2019-08-21

**Authors:** Zinah Zamil Al-Ghezi, Kathryn Miranda, Mitzi Nagarkatti, Prakash S. Nagarkatti

**Affiliations:** Department of Pathology, Microbiology and Immunology, University of South Carolina School of Medicine, Columbia, SC, United States

**Keywords:** multiple sclerosis, EAE, THC, CBD, CB1, CB2, miR-21a-5p

## Abstract

Multiple sclerosis (MS) is a chronic and disabling disorder of the central nervous system (CNS) characterized by neuroinflammation leading to demyelination. Recently a combination of Δ9-tetrahydrocannabinol (THC) and Cannabidiol (CBD) extracted from Cannabis has been approved in many parts of the world to treat MS-related spasticity. THC+CBD combination was also shown to suppresses neuroinflammation, although the mechanisms remain to be further elucidated. In the current study, we demonstrate that THC+CBD combination therapy (10 mg/kg each) but not THC or CBD alone, attenuates murine experimental autoimmune encephalomyelitis (EAE) by reducing neuroinflammation and suppression of Th17 and Th1 cells. These effects were mediated through CB1 and CB2 receptors inasmuch as, THC+CBD failed to ameliorate EAE in mice deficient in CB1 and CB2. THC+CBD treatment also caused a decrease in the levels of brain infiltrating CD4+ T cells and pro-inflammatory molecules (IL-17, INF-γ, TNF-α, IL-1β, IL-6, and TBX21), while increasing anti-inflammatory phenotype such as FoxP3, STAT5b, IL-4, IL-10, and TGF-β. Also, the brain-derived cells showed increased apoptosis along with decreased percentage in G0/G1 phase with increased percentage in G2/M phase of cell cycle. miRNA microarray analysis of brain-derived CD4+ T cells revealed that THC+CBD treatment significantly down-regulated miR-21a-5p, miR-31-5p, miR-122-5p, miR-146a-5p, miR-150-5p, miR-155-5p, and miR-27b-5p while upregulating miR-706-5p and miR-7116. Pathway analysis showed that majority of the down-regulated miRs targeted molecules involved in cycle arrest and apoptosis such as CDKN2A, BCL2L11, and CCNG1, as well as anti-inflammatory molecules such as SOCS1 and FoxP3. Additionally, transfection studies involving miR-21 and use of *Mir21*^−/−^ mice suggested that while this miR plays a critical role in EAE, additional miRs may also be involved in THC+CBD-mediated attenuation of EAE. Collectively, this study suggests that combination of THC+CBD suppresses neuroinflammation and attenuates clinical EAE development and that this effect is associated with changes in miRNA profile in brain-infiltrating cells.

## Introduction

Multiple Sclerosis (MS) is chronic autoimmune disease that affects the central nervous system (CNS) (1–3). The incidence of MS is higher in women who are affected twice as often as men (4). Although the exact etiology of MS remains obscure, observational research has suggested that genetic and environmental factors may cause the onset and progression of the disease (5). Typically, MS is regarded as a T cell mediated autoimmune disorder, primarily driven by inflammatory Th1 and Th17 cells (5, 6). When autoreactive T-lymphocytes cross the blood brain barrier (BBB) and enter the central nervous system, they initiate local inflammation that results in demyelination, gliotic scarring, and axonal damage (7).

Cannabinoids extracted from marijuana (*Cannabis sativa*), as well as synthetic forms have been well-characterized for their anti-inflammatory properties (8). Cannabinoids have also been shown to ameliorate spasticity and neuropathic pain in MS patients (9, 10). It is for this reason, a combination of Δ9—tetrahydrocannabinol (THC) and Cannabidiol (CBD) has been approved as a drug (Sativex) in several countries including Europe, Australia and Canada (11). THC and CBD combination was tested recently in animal models of MS and was found to suppress neuroinflammation (12, 13). However, the precise mechanisms, such as the role of miRNA, in the efficacy of such combination treatment remain to be elucidated further. THC is well-known for its psychoactive properties. It acts through CB1 receptors primarily expressed in the CNS and CB2 expressed predominantly on immune cells (14). Our laboratory and others have shown that THC increases anti-inflammatory and decreases pro-inflammatory cytokine production (15–18). THC also mediates apoptosis in T cell driven inflammation, increases FoxP3^+^ Tregs through miRNA induction and epigenetic modifications (16, 17). On the other hand, CBD is a non-psychoactive phytocannabinoid, has also been shown to exhibit anti-inflammatory properties (19). CBD has recently been approved by US FDA as a drug to treat epilepsy (14). Unlike THC, CBD does not bind and activate CB1 and CB2 receptors but can act as a negative allosteric modulator of CB receptors (20). Also, CBD has been shown to activate other receptors such as GPR55, TRPV1, or 5-HT1a (21–23). Thus, it is possible that a combination of THC+CBD may be more effective in treating inflammation by targeting both cannabinoid CB1 and CB2 receptors as well as other potential receptors such as GPR55, TRPV1, or 5-HT1a.

MicroRNAs (miRNA, miR) are a class of short non-coding single-stranded RNAs 19-24 nucleotides in length, involved in the post-transcriptional regulation of gene expression (3, 24, 25) miRNAs exert their regulatory role when they bind to the 3′ untranslated region (UTR) of target mRNA, eventually causing translational suppression through degradation or sequestration of mRNA (26). Several studies have detected the involvement of circulating miRNAs in physiological and pathological processes and identified them as potential biomarkers, therapeutic agents, or drug targets (27). Numerous miRNAs were found to be differentially expressed in patients with MS compared with controls and to have the potential to be used as diagnostic biomarkers or predictors of drug-response (28). Additionally, recent studies have shown crucial roles of specific miRNAs in controlling oligodendrocyte (OL) differentiation and myelination (29). Dysregulation of miRNAs contributes to the pathogenesis of demyelinating diseases (30). Moreover, new patents of miRNAs also provide new strategies for gene therapy and miRNA-drug development for demyelinating diseases, especially MS (31). Our lab has previously shown that cannabinoids can suppress inflammation in the periphery through regulation of miRNA (3, 15, 32). However, whether cannabinoids can alter the expression of miRs in the brain-infiltrating cells during EAE and whether such miRs contribute toward suppression of neuroinflammation has not been investigated. In the current study, we used the combination of cannabinoids, THC and CBD, to address the potential ability of these components in ameliorating the symptoms and the progression of the disease in the EAE model, a murine model of MS. We demonstrate for the first time that the neuroprotective and anti-inflammatory properties of THC+CBD can be attributed to their ability to induce cell cycle arrest and apoptosis in activated T cells as well as a switch of cytokines from pro-inflammatory to anti-inflammatory, through altered expression of miRNAs.

## Materials and Methods

### Mice

C57BL/6 female mice aged 6–8-week-old and *Mir21*^−/−^ mice were purchased from the Jackson Laboratory (Bar Harbor, ME). CB1^−/−^CB2^−/−^ mice were bred in-house. Mice were housed in a specific-pathogen-free facility at the University of South Carolina School of Medicine. All animal experiments were ethically performed according to the NIH guidelines and protocols approved by the University of South Carolina Institutional Animal Care and Use Committee.

### Reagents

The reagents used in this study were purchased as described: THC and CBD from Cayman Chemical (Michigan, USA), myelin oligodendrocyte glycoprotein (MOG_35−55_) peptide H-MEVGWYRSPFSRVVHLYRNGK-OH (PolyPeptide Laboratories, San Diego, CA, USA). *Mycobacterium tuberculosis* (strain H37Ra) (BD, Franklin Lakes, NJ, USA), complete Freund's adjuvant (Fisher, Hampton, NH, USA), Pertussis toxin (List Biological Laboratories, Campbell, CA, USA), Percoll, GE Healthcare Life Sciences (Pittsburgh, PA, USA); Neural Tissue Dissociation Kit (P) (Miltenyi Biotech, Auburn, CA, USA), RBC lysis buffer (Sigma-Aldrich, St. Louis, MO, USA), RPMI 1640, l-glutamine, HEPES, phosphate-buffered saline, and fetal bovine serum (VWR, West Chester, PA, USA), ELISA Max Kits IL-10, IL-17A, IFN-γ, IL-6, IL-1β, TNF-α, and TGF-β and FITC Annexin V/-PI apoptosis kit (Biolegend, San Diego, CA). EasySep PE selection kit (Stemcell Technologies, Cambridge, MA, USA), Propidium Iodide (PI)/RNase Staining Solution (Cell Signaling Technology, Danvers, MA, USA), miRNeasy Mini Kit, miScript II RT Kit and miRNAs primers (Qiagen, Valencia, CA), mRNAs primers (Integrated DNA technologies, Coralville, IA, USA) and SsoAdvanced™ Universal SYBR® Green Supermix (Bio-Rad, Hercules, CA, USA).

### EAE Induction, Cannabinoid Administration, and Clinical Assessment

EAE was induced in female C57BL/6 mice (6–8 weeks old) through subcutaneous (s.c.) immunization in the hind flank with 100 μl of 150 μg MOG_35−55_ peptide (PolyPeptide Laboratories San Diego, CA, USA) emulsified in complete Freund's adjuvant (CFA) (Fisher, Hampton, NH, USA) containing 8 mg/ml killed Mycobacterium tuberculosis (strain H37Ra) (BD, Franklin Lakes, NJ, USA), as described previously (32, 33) Following immunization, 200 ng of pertussis toxin (List Biological Laboratories, Campbell, CA, USA) was given to the mice by intraperitoneal injection on day 0, followed by 400 ng on day 2. Control mice received CFA+PTX but not MOG. To study the effect of THC+CBD treatment mice were randomized and treated with 10 mg/kg each THC and CBD or vehicle (2% dimethyl sulfoxide (DMSO) + 20% EtOH) diluted with sterile 1X PBS i.p. starting on day 10 after immunization and this treatment continued every day until the end of the experiment. Monitoring the animals and recording the clinical scores were done on a daily basis during the experiment. The mean of the score was calculated for each group every day. Clinical scores were recorded as follow: 0, healthy; 1, flat tail; 2, partial paralysis of hind limbs; 3, complete paralysis of hind limbs or partial hind and front limb paralysis; 4, tetraparalysis; 5, moribund; 6, death (34). Mice were provided daily with food and water (Boost and Hydrogel) in the cage floor after appearance of symptoms to ensure access to essential nourishment.

### Histopathology

Perfused spinal cord tissues were isolated at 15 days post MOG immunization. Tissues were immersed in 4% paraformaldehyde for 24 h. Then paraffin blocks were prepared. Microtome sections (7 μm) were cut, and tissue sections were stained with Luxol Fast Blue (LFB) for detection of demyelination, in addition to haemotoxylin and eosin (H&E) staining for visualization of cellular infiltration. The images were acquired by Cytation 5 imaging reader (BioTek).

### Isolation of Immune Cells

On day 15 post MOG immunization, inguinal lymph nodes (iLN) were excised from, EAE+Vehicle and EAE+(THC+CBD) prior to perfusion and were processed immediately to prepare single-cell suspensions. Then, mice were perfused slowly with 10 mL heparinized PBS to get rid of contaminated blood. Whole brain tissues were isolated then homogenized separately into a single-cell suspension by using the Neural Tissue Dissociation Kit (P) (Miltenyi Biotech, Auburn, CA, USA) and red blood cell lysis buffer (Sigma-Aldrich, St. Louis, MO, USA). Mononuclear cells (MNC) from whole brain homogenates were then isolated by centrifugation in media containing 33% (v/v) isotonic Percoll in FACS buffer (1X PBS, 2% heat-inactivated fetal bovine serum) (GE Healthcare Life Sciences, Pittsburgh, PA, USA). Cells were immediately counted and processed for further assays.

### Cell Culture

Brain MNCs and splenocytes cells were cultured for 24 h in complete RPMI 1640 media supplemented with 10% heat-inactivated fetal bovine serum, 10 mM l-glutamine, 10 mM HEPES, 50 μM β-mercaptoethanol (Sigma-Aldrich St. Louis, MO, USA), and 100 μg/ml penicillin/streptomycin at 37°C, 5% CO_2_, 95% humidity (32). Cell culture supernatants were collected for ELISA and/or cells were processed for flow cytometry, apoptosis and cell cycle assays.

### Detection of Cytokines

Brain and iLN were isolated from EAE+VEH and EAE+(THC+CBD) mice and processed to obtain single-cell suspensions, and 1 ×10^6^ cells were cultured for 24 h at 37°C, 5% CO_2_, 95% humidity as described (32). Cell culture supernatants were processed to detect interferon-γ (IFNγ), interleukin-17A (IL-17A), interleukin-6 (IL-6), Tumor Necrosis Factor-α (TNFα), interleukin 1β (IL-1 β), interleukin-10 (IL-10) and transforming growth factor-β (TGF-β) using ELISA kits following the manufacturer's instructions (BioLegend, San Diego, CA). Absorbance at 450 nm was read on a plate reader and concentrations were calculated using standard curves.

### Antibodies and Flow Cytometry

Cells were stained with fluorochrome-conjugated antibodies and analyzed via BD FACS Celesta (San Jose, CA) to determine phenotypes of infiltrating brain mononuclear cells. Antibodies used: fluorescein isothiocyanate (FITC) -conjugated anti-CD3 (clone: 145-2C11), Brilliant Violet (BV785)-conjugated anti-CD4 (clone: GK 1.5), from Biolegend (San Diego, CA).

### Detection of (THC+CBD)-Induced Apoptosis in Brain MNCs

To determine if (THC+CBD) induces apoptosis in brain MNCs, cells were purified and cultured as described (32). After 24 h incubation, cells were collected and washed twice with ice-cold 1X PBS, and then resuspended in Annexin V Binding Buffer at a concentration of 0.25–1.0 ×10^7^ cells/ml. Next, 100 μl of cell suspension was transferred to a 5 ml flow tube. Next, 5 μl of FITC Annexin V and 10 μl of Propidium Iodide Solution was added. The cells were gently vortexed and incubate for 15 min at room temperature in the dark. Finally, 400 μl of Annexin V Binding Buffer was added to each tube and analyzed by flow cytometry (BioLegend, San Diego, CA, USA).

### Cell Cycle Analysis

Brain MNCs were cultured as described (32). Cells were collected and stained with the PI/RNAs staining following the manufacturer's instructions (Cell Signaling Technology, Danvers, MA, USA). The data were acquired by flow cytometry and analyzed with ModFit LT 3.3 (Verity Software House, Topsham, ME) after debris and doublets were gated out.

### CD4+ T Cell Selection

Brain MNCs were labeled with Phycoerythrin (PE)-conjugated anti-CD4 (Clone: GK 1.5) antibody (BioLegend, San Diego, CA) then immunomagnetically selected with EasySep PE-positive selection kit according to the manufacturer instructions (StemCell Technologies, Vancouver, BC). After selection, the purity of selected CD4 was measured by flow cytometry which was routinely >90%. CD4^+^ T cells were lysed in Qiazol and stored at −80°C until RNA isolation (Qiagen).

### RNA Isolation and cDNA Synthesis

Total RNA was purified from brain CD4^+^ T cells by using miRNeasy micro kit according to the manufacturer instructions and the concentration and purity of RNA were determined using the NanoDrop 2000 spectrophotometer from Thermo Scientific (Wilmington, DE). Next, the expression profiling of miRNAs using the Affymetrix GeneChip miRNA 4.0 array platform was performed as previously described (35). To validate miRNAs expression, the miScript cDNA synthesis kit used followed by quantitative real-time polymerase chain reaction (qRT-PCR) using the miScript SYBR Green PCR kit. Fold change of the interested miRNAs was determined using the 2^−ΔΔ*Ct*^ method and expressed relatively to Snord96a (Bio-Rad, Hercules, CA, USA). Validation of target genes expression, primers were purchased (Integrated DNA technologies, Coralville, IA, USA) and quantitative real-time polymerase chain reaction (qRT-PCR) was performed using SsoAdvanced universal SYBR Green supermix (Bio-Rad, Hercules, CA, USA). Fold change of the interested mRNAs was determined using the 2^−ΔΔ*Ct*^ method and expressed relative to GAPDH.

### Transfection With miR-21a-5p Mimic and Inhibitor

Splenic CD4+ T cells were purified by using the EasySep PE selection kit. The purity of the isolated cells was confirmed to be 97% CD4+ T cells by flow cytometry. Then cells were maintained for 24 h in complete RPMI 1640 media supplemented with 10% heat-inactivated fetal bovine serum, 10 mM l-glutamine, 10 mM HEPES, 50 μM β-mercaptoethanol, and 100 μg/ml penicillin/streptomycin at 37°C and 5% CO_2_ (16). Cells were seeded at 2 ×10^5^ cells/well in a 24-well plate and transfected for 24 h with mock control or 40 nM synthetic mimic or inhibitor oligonucleotides using HiPerFect transfection reagent (Qiagen, Germantown, MD) according to the manufacturer's instructions. Total RNA and protein were extracted for analysis.

### Statistical Analysis

We performed statistical analysis using GraphPad Prism 8 (GraphPad Inc, La Jolla, CA). The data shown in this study represent at least three independent experiments to ensure consistency of findings. The statistical differences between groups were calculated using Student's *t*-test for paired analyses or one- or two-way ANOVA for multiple group analyses. Mann–Whitney *U*-test was performed to evaluate the extended clinical scoring in EAE mice, as described (32, 36). Statistical tests with *post hoc* tests are indicated in each figure legend. A *p-*value of ≤ 0.05 was considered significant.

## Results

### Combination of THC and CBD Attenuate the Development of EAE

**Figure 1:**
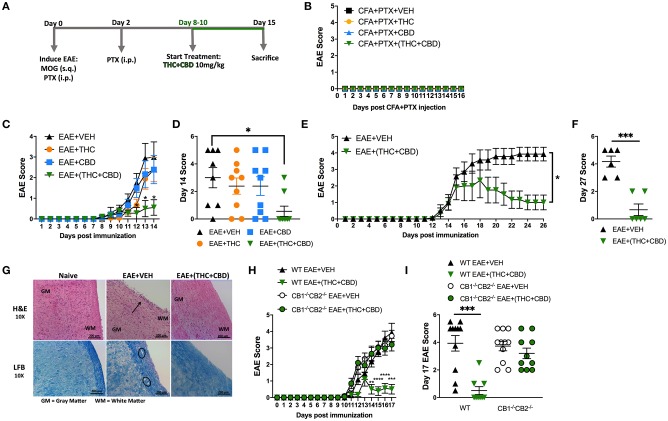
Combination of THC+CBD attenuates EAE by suppressing neuroinflammation. EAE was induced in C57BL/6 mice using CFA+PTX+MOG, as described in Methods. These mice were treated with cannabinoids and the mice were studied for clinical signs of paralysis and neuroinflammation. **(A)** Experimental timeline. EAE mice were treated daily with THC (10 mg/kg), CBD (10 mg/kg), or a combination of THC+CBD (10 mg/kg each) starting at 8–10 days after MOG immunization. **(B)** Controls consisting of mice that received CFA+PTX only that received cannabinoids. **(C,D)** Clinical scoring of EAE symptoms in mice treated with Veh, THC, CBD, or THC+CBD. **(E,F)** EAE scoring in an extended experiment until day 27 in Veh vs. THC+CBD treated EAE mice. **(G)** Representative H&E images and LFB staining in spinal cord tissues to detect cellular infiltration and demyelination, respectively. **(H,I)** EAE scoring in WT or CB1^−/−^CB2^−/−^ double knockout mice treated with either Veh or THC+CBD. Data presented are mean ± SEM. For **(B,C,H)**, significance was determined by two-way ANOVA with a Dunnett *post hoc* test. For **(D,I)**, one-way ANOVA with a Dunnett *post hoc* test was used. For **(E)** Mann-Whitney U test was performed excluding scoring values before initiation of treatment. For **(F)**, an unpaired two-tailed *T*-test was performed. For **(J)**, one-way ANOVA with a Sidak correction was used. ^****^p < 0.0001, ^***^p < 0.001, ^**^p < 0.01, ^*^p < 0.05.

Combination of THC+CBD has been used to treat human MS (37). This treatment is known to decrease not only muscle spasticity but also suppress neuroinflammation (12, 13). To further investigate the mechanisms of suppression of neuroinflammation, we used murine model of EAE. Mice were treated daily with THC alone (10 mg/kg), CBD alone (10 mg/kg), or a combination of THC+CBD (10 mg/kg each) starting at 8–10 days after MOG immunization (Figure 1A). Use of CFA+PTX as a control did not trigger any clinical signs of paralysis and furthermore, treatment of these mice with cannabinoids did not have any effect, thereby showing that the subsequent studies reported on EAE development using MOG was antigen-specific (Figure 1B). Thus, in all subsequent experiments, we used CFA+PTX+MOG to induce EAE and study the effect of cannabinoids. The combination THC+CBD treatment resulted in attenuation of the clinical symptoms of EAE vs. mice treated with Vehicle (VEH) (Figures 1C,D). Also, treatment with THC or CBD alone, at the doses tested, failed to cause significant suppression of clinical symptoms. On day 14, the clinical scores were significantly reduced only in THC+CBD group but not in THC or CBD alone groups (Figure 1D). These results indicated that the combination of THC+CBD was effective to treat mice with EAE. Based on these data, we focused our subsequent studies to combination treatment only. Thus, an extension of the experiment until day 27 demonstrated that THC+CBD treatment was highly effective long-term at reducing clinical signs of EAE (Figures 1E,F).

Next, we performed histological analysis on spinal cord tissues harvested at day 15. The spinal cord tissues from EAE+VEH mice showed elevated cellular infiltration vs. Naïve mice when stained with H&E (Figure 1G). Also, extensive demyelination was observed in the white matter area with LFB staining in EAE+VEH vs. Naïve mice (Figure 1G). Both cellular infiltration and demyelination were reduced in spinal cord tissue of EAE+(THC+CBD) mice (Figure 1F). To test the role of CB1 and CB2 receptors in our model, we induced EAE in both wild-type (WT) and CB1^−/−^CB2^−/−^ double-knockout mice, and then treated with THC+CBD. Absence of cannabinoid receptors resulted in the inability of THC+CBD to reduce clinical scores of EAE (Figures 1H,I).

**Figure 2:**
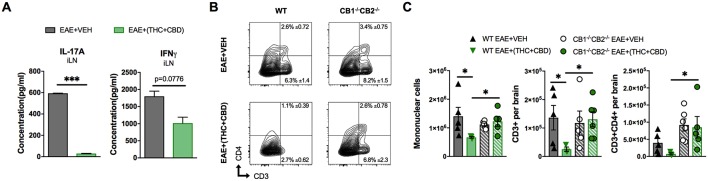
T cell population and phenotypic changes of WT and CB1^−/−^CB2 ^−/−^ EAE mice treated with vehicle or THC+CBD. As shown in Figure 1, EAE was induced in naïve mice then mice were treated with Veh or THC+CBD when symptoms appeared. Inguinal lymph nodes and brain MNCs were collected at the peak of disease (Day 15 post immunization). **(A)** IL-17A and IFNγ concentration in inguinal lymph node 24 h culture supernatant measured by ELISA. **(B)** Representative flow cytometry contour plots of encephalitogenic T cells. **(C)** Quantification of total MNCs, CD3+ T cells, and CD3+CD4+ Th cells per brain. Data presented are mean ± SEM. ^***^p < 0.001, ^*^p < 0.05 by unpaired two-tailed *T*-test **(A)**, or Kruskal-Wallis test **(C)**.

Cell culture supernatants were isolated from draining iLN cells isolated at the peak of the disease. The cells were cultured at equivalent cellular density for 24 h and supernatants were assessed for the Th17 and Th1 pro-inflammatory cytokines, IL-17A and IFNγ, respectively. THC+CBD treatment reduced production of IL-17A and IFNγ in iLN (Figure 2A). Additionally, flow cytometry analysis of encephalitogenic mononuclear cells (MNC) isolated from brain tissue showed decreases in the populations of total MNCs, CD3+ T cells, and of CD3+CD4+ Th cells in the EAE+(THC+CBD) group when compared to other experimental groups (Figures 2B,C). Use of CB1^−/−^CB2^−/−^ double-knockout mice showed that the effect of THC+CBD in decreasing neuroinflammation was mediated through these cannabinoid receptors (Figures 2B,C) because THC+CBD was ineffective in these mice.

### miRNA Analysis of THC+CBD Treated EAE Mice

**Figure 3:**
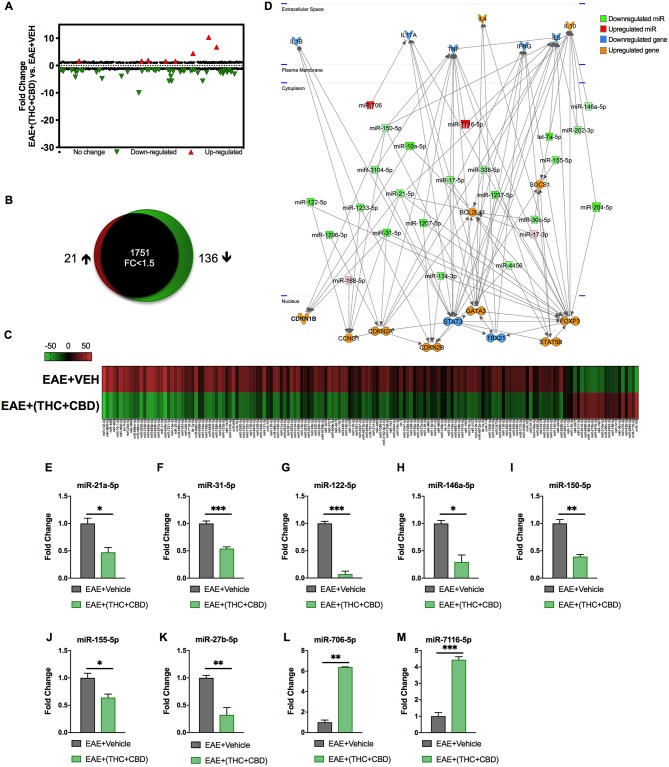
Differentially expressed miRNAs in brain-infiltrating CD4+ T cells upon THC+CBD treatment in EAE mice. Total RNA was isolated and pooled from CD4+ T cells obtained from the brains of EAE mice post-treated with vehicle (*n* = 5) or THC+CBD (*n* = 5) on day 15. MicroRNA microarray expression levels of differentially expressed miRNAs were generated. **(A)** The fold change distribution of all 1,908 miRNAs tested. **(B)** Proportional Venn diagram illustrating fold change of miRNAs that were >1.5- fold dysregulated following treatment with THC+CBD in EAE mice. **(C)** Heat map of the 157 dysregulated miRNAs. The color scale denotes those miRNAs that were upregulated (red) and downregulated (green). **(D)** Pathway analysis of miRs mediating dysregulation in gene expression following THC+CBD treatment. **(E–M)** Expression levels of selected upregulated and downregulated miRNAs were validated by qRT-PCR using Snord96a as a small RNA endogenous control. Data presented are mean ± SEM. ^***^p < 0.001, ^**^p < 0.01, ^*^p < 0.05 by unpaired two-tailed *T*-test.

Because miRNAs play an important role in autoimmune diseases and neuroinflammation (38, 39), we investigated the role of miRNA in the THC+CBD-induced attenuation of neuroinflammation in EAE mice. To that end, brain CD4+ T cells were isolated from mice treated with THC+CBD or vehicle as described earlier and used for miRNA microarray analysis. Of approximately 2000 miRNAs tested, 157 miRNAs were differentially expressed (Fold change > ± 1.5) (Figure 3A). Proportional Venn diagram was generated to represent the fold change of the miRNAs that were up- or down-regulated following treatment with THC+CBD in EAE mice (Figure 3B). A heat map generated showed different expression profile of miRNAs in the experimental groups (Figure 3C). Pathway analysis of the differentially expressed miRNAs was performed with Ingenuity Pathway Analysis (IPA, Qiagen) and showed interaction with cell cycle, apoptosis, and T cell polarization molecules (Figure 3D). The microarray data and pathway analysis indicated several miRNAs that have been previously involved in the pathogenicity of MS such as miR-31,−21a,−146a,−155, and−33 (40). Quantitative RT-PCR validated that THC+CBD treatment led to downregulation miR-21a-5p, miR-31-5p, miR-122-5p, miR-146a-5p, miR-150-5p, miR-155-5p, and miR-27b-5p (Figures 3E–K). These miRNAs were found to directly target IL-10, FoxP3, SOCS1, Bcl2L11, and CCNG1 (Figure 3D and Tables 1 and 2). THC+CBD treatment increased expression of miR-706-5p and miR-7116 (Figures 3L,M). The IL-17A gene was one of the hallmark genes that is targeted by miR-706-5p (Figure 3D and Table 1). Genes encoding TNF-α and IL-6 were found to be targeted by miR-7116-5p (Figure 3D and Tables 1 and 2). These genes play a pivotal role in EAE progression. Putative 3' UTR targeting was analyzed for each miRNA-mRNA pairing using TargetScan alignment tools and microRNA.org (Table 1). Collectively, these data indicated that microRNA may have an integral role in the ameliorative effect of THC+CBD in EAE mice.

**Table 1:** 3′UTR alignments and scores of miRs and their target genes.

**CCNG1**		
3^′^ guuugugguaacagUGUGAGGu 5^′^ mmu-miR-122	mirSVR score:	−1.0549
| | | | | | |	PhastCons score:	0.5536
1796:5^′^ uuaggcuugauaaaACACUCCa 3^′^ Ccng1		
3^′^ uuggguacCUUAAG– – –UCAAGAGu 5^′^ mmu-miR-146a	mirSVR score:	−0.1505
| | | | | | | | | | | |	PhastCons score:	0.4986
921:5^′^ agaacgaaGAACUCCCAAGUUCUCu 3^′^ Ccng1		
3^′^ cgcCUUGAAUCGGUGACACUu 5^′^ mmu-miR-27a	mirSVR score:	−1.0267
| | | | | | | | | | | |	PhastCons score:	0.5944
1306:5^′^ uguGAAAUAA – – AACUGUGAa 3^′^ Ccng1		
3^′^ guguuugguaauacACGACGAu 5^′^ mmu-miR-15a	mirSVR score:	−0.4111
| | | | | | |	PhastCons score:	0.5536
1682:5^′^ aaauuugucagaacUGCUGCUu 3^′^ Ccng1		
3^′^ guGACCAUGUUCCCAACCCUCu 5^′^ mmu-miR-150	mirSVR score:	−0.2496
| | | | | | | | | | | | | |	PhastCons score:	0.5917
1226:5^′^ ucCUGAUUCUAAGCUUGGGAGa 3^′^ Ccng1		
**CDKN1b**		
3^′^ aguUGUAGUCAGACUAUUCGAu 5^′^ mmu-miR-21	mirSVR score:	−0.5432
| :| : | | | :| | | | | | | |	PhastCons score:	0.537
1093:5^′^ cuuAUAAUAGUUU–AUAAGCUc 3^′^ Cdkn1b		
3^′^ gugUUUA–AGC–CUA–GAUGUCCCAu 5^′^ mmu-miR-10a	mirSVR score:	−0.1534
:| | | | | | | | | | | | | | |	PhastCons score:	0.537
1236:5^′^ aagGAAUAUAGAGAUGGCACAGGGUu 3^′^ Cdkn1b		
3^′^ gugACCAUGUU–CCCAACCCUCu 5^′^ mmu-miR-150	mirSVR score:	−0.0062
| | | | | | | | | | | | |	PhastCons score:	0.5328
1376:5^′^ aagUUGUCGAAUUGGAUGGGAGu 3^′^ Cdkn1b		
3^′^ ugGGGAUAGUGUUAAUCGUAAUu 5^′^ mmu-miR-155	mirSVR score:	−0.0055
:::| | | :: | | :| | | | | |	PhastCons score:	0.5372
1811:5^′^ caUUUUAGAAUGUUUGGCAUUAu 3^′^ Cdkn1b		
3^′^ gucGAUACGGUCGUAGAACGGa 5^′^ mmu-miR-31	mirSVR score:	−0.0062
| | | | | | | | | | | | |	PhastCons score:	0.5482
232:5^′^ uucCCAAGCAAG–AACUUGCCa 3^′^ Cdkn1b		
**Bcl2l11**		
3^′^ gugagucguGGUCCUAUAACAa 5^′^ mmu-miR-338-5p	mirSVR score:	−0.0008
| | | :| | | | | |	PhastCons score:	0.5603
803:5^′^ ggguaucuuCAAGUGUAUUGUg 3^′^ Bcl2l11		
3^′^ aguUGUAGUCAGACUAUUCGAu 5^′^ mmu-miR-21	mirSVR score:	−0.1597
| :| | :| | | | | | | | | |	PhastCons score:	0.546
1814:5^′^ uguAUAUUA–CCU–AUAAGCUu 3^′^ Bcl2l11		
3^′^ guguuuaagccuagauGUCCCAu 5^′^ mmu-miR-10a	mirSVR score:	−0.0017
| | | | | |	PhastCons score:	0.5603
785:5^′^ augcgcagcuucagccCAGGGUa 3^′^ Bcl2l11		
**IL10**		
3^′^ gugaccauguucccaACCCUCu 5^′^ mmu-miR-150	mirSVR score:	−0.0295
| | | | | |	PhastCons score:	0.6418
290:5^′^ gauuauauuauaugaUGGGAGg 3^′^ Il10		
3^′^ uuGGGUACCUUAAGUCAAGAgu 5^′^ mmu-miR-146a	mirSVR score:	−0.586
| | | | | | | | | | | | |	PhastCons score:	0.7581
578:5^′^ acCACCUAAAAUU - AGUUCUaa 3^′^ Il10		
3^′^ aguuguagucagacuAUUCGAu 5^′^ mmu-miR-21	mirSVR score:	−0.155
| | | | | |	PhastCons score:	0.6617
233:5^′^ uuuuuaaccuguguuUAAGCUg 3^′^ Il10		
3^′^ uccGUAUCCUACUGUUUCCCUu 5^′^ mmu-miR-204	mirSVR score:	−1.2036
:| | | | | | :| | | | | | |	PhastCons score:	0.6418
270:5^′^ cuuUAUAGUAU -UUAAAGGGAg 3^′^ Il10		
3^′^ uuGGGUACCUUAAGUCAAGAgu 5^′^ mmu-miR-146a	mirSVR score:	−0.586
| | | | | | | | | | | | |	PhastCons score:	0.7581
578:5^′^ acCACCUAAAAUU -AGUUCUaa 3^′^ Il10		
3^′^ cgccuUGAAUCG – GUGACACUu 5^′^ mmu-miR-27a	mirSVR score:	−0.0968
| | | | | | | | | | | | |	PhastCons score:	0.6709
165:5^′^ uauucACUGAGCUUCUCUGUGAa 3^′^ Il10		
**Foxp3**		
3^′^ uccguaUCCUACUGUUUC-CCUu 5^′^ mmu-miR-204	mirSVR score:	−0.194
| | | | | | | | | | | |	PhastCons score:	0.5948
896:5^′^ gaucccAGCAGGAGAAAGCGGAu 3^′^ Foxp3		
3^′^ gucgauacggucguaGAACGGa 5^′^ mmu-miR-31	mirSVR score:	−0.6807
| | | | | |	PhastCons score:	0.7094
695:5^′^ guaccccacgucucaCUUGCCa 3^′^ Foxp3		
3^′^ guGUUUGGUAAUACAC-GACGAu 5^′^ mmu-miR-15a	mirSVR score:	−0.1344
| :::| | | | | | | | | | | | |	PhastCons score:	0.5291
97:5^′^ acCGGGCGAUGAUGUGCCUGCUa 3^′^ Foxp3		
3^′^ guuugugguaacaguGUGAGGu 5^′^ mmu-miR-122	mirSVR score:	−0.0057
| | | | | |	PhastCons score:	0.5172
1243:5^′^ guauguccuucccucCACUCCa 3^′^ Foxp3		
**IL17a**		
3^′^ aaaaaacucuguccCAAAGAGa 5^′^ mmu-miR-706	mirSVR score:	−0.6735
| | | | | | |	PhastCons score:	0.4105
23:5^′^ uaagaaacccccacGUUUCUCa 3^′^ Il17a		
**TNF**		
5^′^…CCCAGUGUGGGAAGCUGUCUUCA… (Position 529-535)	Targetscan context score:−0.17	
| | | | | | |		
3^′^ AAAAAAAAGGACUACAGAAGU mmu-miR-7116-5p	Targetscan context++ score percentile: 86	
**IL6**		
5^′^…UUAUAAUGUUUAGAC-UGUCUUCA… (Position 390-397)	Targetscan context score:-0.57	
| | | | | | | | | |		
3^′^ AAAAAAAAGGACUACAGAAGU mmu-miR-7116-5p	Targetscan context++ score percentile: 99	

**Table 2:** miRNAs with their seed sequences and fold changes.

**MicroRNA identification**	**MicroRNA sequence**	**Seed sequence**	**Fold change**
mmu-miR-7116-5p	UGAAGACAUCAGGAAAAAAAA	GAAGACA	6.8
mmu-miR-706	AGAGAAACCCUGUCUCAAAAAA	GAGAAAC	10.4
mmu-miR-122-5p	UGGAGUGUGACAAUGGUGUUUG	GGAGUGU	−25.8
mmu-miR-21a-5p	UAGCUUAUCAGACUGAUGUUGA	AGCUUAU	−2.4
mmu-miR-155-5p	UUAAUGCUAAUUGUGAUAGGGGU	UAAUGCU	−2.5
mmu-miR-338-5p	AACAAUAUCCUGGUGCUGAGUG	ACAAUAU	−1.8
mmu-miR-146a-5p	UGAGAACUGAAUUCCAUGGGUU	GAGAACU	−1.7
mmu-miR-31-5p	AGGCAAGAUGCUGGCAUAGCUG	GGCAAGA	−4.7
mmu-miR-27-5p	AGGGCUUAGCUGCUUGUGAGCA	GGGCUUA	−1.8
Mmu-miR-204-5p	UUCCCUUUGUCAUCCUAUGCCU	UCCCUUU	−6.5

### Cytokine Expression at Gene and Protein Levels in EAE Brain MNCs

**Figure 4:**
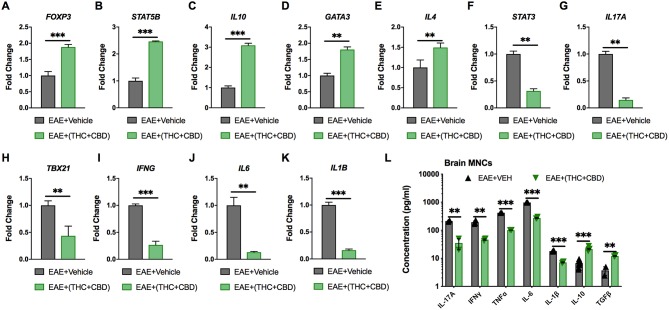
Expression of miRNA target genes involved in Th cell polarization. As described in Figures 2, 3, CD4+ T cells or total MNCs were isolated from brains of EAE mice on day 15 post immunization. For **(A–K)**, RNA was extracted from CD4+ T cells and used for miR target gene validation. For **(L)**, total MNCs were cultured for 24 h and supernatants were collected for cytokine analysis. **(A–K)** qRT-PCR validation of the target genes **(A)** Foxp3, **(B)** STAT5B, **(C)** IL-10, **(D)** GATA3, **(E)** IL-4 **(F)** STAT3, **(G)** IL-17A, **(H)** TBX21, **(I)** IFN-γ, **(J)** IL-6, and **(K)** IL-1β using GAPDH as endogenous control. **(L)** IL-17A, IFN-γ, TNF-α, IL-6 IL-1β, IL-10, and TGF-β concentration measured in MNC culture supernatants by ELISA. Data are expressed as the mean ± S.E.M. and statistical significance is indicated as ^***^p < 0.001, ^**^p < 0.01 by unpaired two-tailed *T*-test.

Pathway analysis identified miRNAs that targeted pro-inflammatory and anti-inflammatory cytokines and Th subset transcription factors (Figure 3D). Expression of these target genes was validated by qRT-PCR (Figures 4A–K). Treg related genes *Foxp3, Stat5b*, and *IL10* were upregulated in EAE+(THC+CBD) brain-derived CD4+ T cells (Figures 4A–C). Th2 related genes *Gata3* and *Il4* were also upregulated in CD4+ T cells following treatment (Figures 4D,E). Conversely, Th17 related genes *Stat3* and *Il17a* were downregulated following THC+CBD treatment (Figures 4F,G). Likewise, Th1 related genes *Tbx21* (encoding Tbet) and *Ifng* were downregulated in EAE+(THC+CBD) (Figures 4H,I). In addition, pro-inflammatory cytokines *Il6* and *Il1b* were downregulated (Figures 4J,K). Cell culture supernatant of mononuclear cells from brain was used to evaluate cytokine production. In accordance with gene expression changes, IL-17A, IFNγ, TNFα, IL-6, and IL-1β production was reduced, while IL-10 and TGFβ production was increased in MNC supernatant from EAE+(THC+CBD) mice (Figure 4L).

### Detection of Cell Cycle Arrest/Apoptosis in Brain MNCs

**Figure 5:**
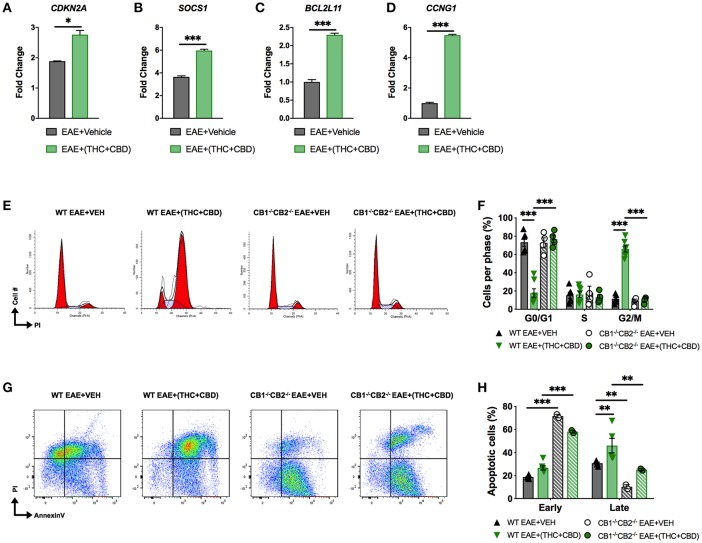
THC+CBD treatment induces cell cycle arrest / apoptosis in brain MNCs. As shown in Figure 1, EAE was induced in naïve mice and treatment with Veh or THC+CBD was initiated at the onset of symptoms. CD4+ T cells and total MNCs were isolated from the brains of mice on day 15 post immunization for gene and cell cycle analysis. **(A–D)** qRT-PCR validation of miR target genes involved in cell cycle/apoptosis. **(E)** Representative flow cytometry histograms of PI staining in brain MNCs using ModFit software. **(F)** Cell cycle phase quantification. **(G)** Representative flow cytometry pseudocolor plots of brain MNCs in early apoptosis (AnnexinV^+^ PI^−^) or late apoptosis (AnnexinV^+^PI^+^). **(H)** Quantification of brain MNCs in early or late apoptosis. Data represented are mean ± SEM. ^***^p < 0.001, ^**^p < 0.01, ^*^p < 0.05 by unpaired two-tailed *T*-test **(A–D)**, or two-way ANOVA with a Tukey *post hoc* test **(F,H)**.

miRNA array and pathway analysis also revealed that some pro-apoptotic and cell cycle arrest genes were targeted by downregulated miRs in EAE+(THC+CBD) mice including *CDKN2A, SOCS1, Bcl2L11*, and *CCNG1* (Figure 3D). We validated upregulation of these genes by qRT-PCR (Figures 5A–D). Fold change was expressed relative to GAPDH. The primers used in the study are highlighted in Table 3. In addition, PI staining demonstrated that WT EAE+(THC+CBD) mice, in brain MNCs, had less cells in G0/G1 phase but more cells in G2/M phase of cell cycle when compared to WT EAE+Veh group (Figures 5E,F). We also used a combination staining of Annexin V-FITC with PI double-staining to identify early apoptotic (AnnexinV^+^/PI^−^) and late apoptotic cells (AnnexinV^+^/PI^+^). Late apoptosis was elevated in WT EAE-(THC+CBD) (Figures 5G,H). In some of these experiments, we also used CB1^−/−^CB2^−/−^ double knockout mice to test if the action of THC+CBD was mediated through cannabinoid receptors and we did find that to be true. However, these mice showed some changes in apoptosis when compared to WT mice which can be explained by the fact that in these mice, endocannabinoids were not able to act or that these mice had some compensatory mechanisms acting.

**Table 3:** mRNA quantitative RT-PCR primer sequences.

**Gene**	**Forward**	**Reverse**
IL-10	CCCATTCCTCGTCACGATCTC	TCAGACTGGTTTGGGATAGGTTT
TGF-β	ATGTCACGGTTAGGGGCTC	GGCTTGCATACTGTGCTGTATAG
IL-4	GGTCTCAACCCCCAGCTAGT	GCCGATGATCTCTCTCAAGTGAT
GATA3	CTCGGCCATTCGTACATGGAA	GGATACCTCTGCACCGTAGC
IL-17A	TTTAACTCCCTTGGCGCAAAA	CTTTCCCTCCGCATTGACAC
IFN-γ	TCCTCGCCAGACTCGTTTTC	GTCTTGGGTCATTGCTGGAAG
T-bet	AGCAAGGACGGCGAATGTT	GGGTGGACATATAAGCGGTTC
IL-6	CCAAGAGGTGAGTGCTTCCC	CTGTTGTTCAGACTCTCTCCCT
TNF-α	GGAACACGTCGTGGGATAATG	GGCAGACTTTGGATGCTTCTT
FOXP3	CCCATCCCCAGGAGTCTTG	ACCATGACTAGGGGCACTGTA
SOCS1	CTGCGGCTTCTATTGGGGAC	AAAAGGCAGTCGAAGGTCTCG
BCL2L11	GACAGAACCGCAAGGTAATCC	ACTTGTCACAACTCATGGGTG
CCNG1	ACAACTGACTCTCAGAAACTGC	CATTATCATGGGCCGACTCAAT
CDKN2B	CCCTGCCACCCTTACCAGA	CAGATACCTCGCAATGTCACG
CACUL1	AACACCTCCACCTCCAAGTT	AGACTCGCTCTAAGTGGCTG
CDCA4	GTAGAGGGTTTTGGCACTGTC	TGGGCTCCACTAGCATGTGA
GAPDH	TGGATTTGGACGCATTGGTC	TTTGCACTGGTACGTGTTGAT

### *Mir21^−/−^* Mice Are More Resistant to EAE Than Wild-Type Mice

**Figure 6:**
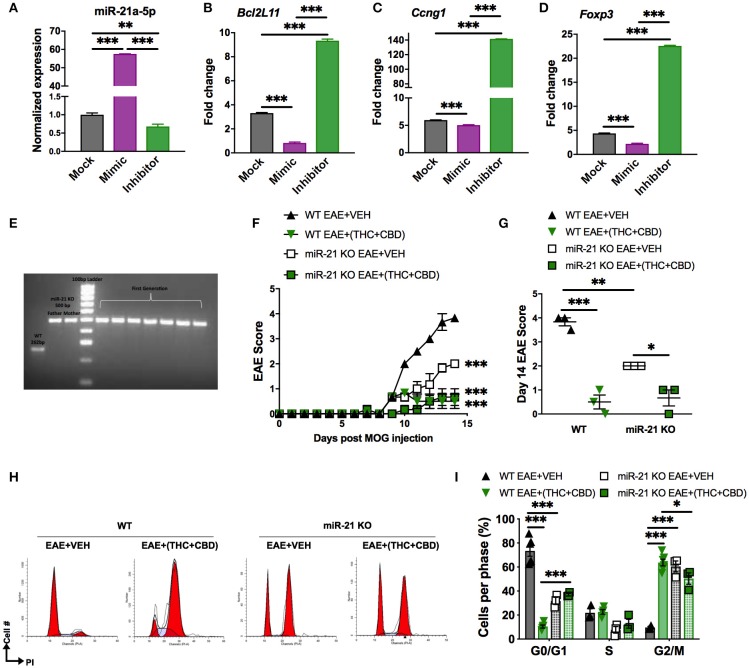
Role of miR-21 downregulation on THC+CBD-mediated amelioration of EAE. For **(A–D)**, miR-21a-5p transfection assays were performed in CD4+ T cells purified from naïve WT C57BL6 mice. Cells were cultured for 24 h and transfected with miR-21a-5p mimic, inhibitor, or mock (transfection reagent only). For **(E,F)** EAE was induced in WT and *Mir21*^−/−^ mice as described in Methods then treated with Veh or THC+CBD upon development of EAE symptoms. **(A–D)** qRT-PCR validation for miR-21 and the target genes. **(E)** Agarose gel electrophoresis of genotyping for the parents and the first generation of *Mir21*^−/−^ mice (“miR-21 KO”). **(F)** EAE scoring in WT and *Mir21*^−/−^ mice. **(G)** Quantification of the clinical scores. **(H)** Representative flow cytometry histograms of brain MNCs stained with PI study cell cycle analysis. **(I)** Quantification of cell cycle phases. Data are expressed as the mean ± S.E.M. and statistical significance is indicated as ^***^p < 0.001, ^**^p < 0.01, ^*^p < 0.05. For **(F)**, statistical significance is vs. WT EAE+Veh. Significance was determined by one- or two-way ANOVA with Tukey *post hoc* corrections.

In our study, we found that THC+CBD treatment downregulated miR-21a-5p expression in brain CD4+ T cells (Figures 3C–E). To further address the role of this miRNA, we performed an *in vitro* miRNA transfection assay in CD4^+^ T cells and used qRT-PCR validation to test for miR-21 and target genes in cells transfected with mock, mimic or inhibitor (Figures 6A–D). The data showed that use of miR-21 mimic led to a decrease in the expression of target genes while inhibitor caused significant induction of the target genes. In addition, we also used mice deficient in miR-21. Genotyping for the parents and the first generation of *Mir21*^−/−^ mice (miR-21 KO) confirmed inactivation of the miR-21 gene (Figure 6E). To test the role of miR-21 in THC+CBD-mediated amelioration of EAE, we induced EAE in WT and *Mir21*^−/−^ mice then treated with THC+CBD when symptoms appeared. The clinical scores revealed that *Mir21*^−/−^ mice had less disease severity when compared with WT EAE mice (Figures 6F,G). Treatment with THC+CBD in *Mir21*^−/−^ mice further reduced clinical symptoms of EAE similar to WT EAE+(THC+CBD) (Figures 6F,G). We performed cell cycle analysis in brain MNCs stained with PI to detect cell cycle by flow cytometry. The EAE-induced *Mir21*^−/−^ mice were more similar to EAE+(THC+CBD) group in that these mice showed less cells in G0/G1 phase and more cells G2/M phase and furthermore, THC+CBD treatment in these mice failed to further cause significant changes in cell cycle thereby showing that THC+CBD-mediated effects on cell cycle are mediated through miR-21 (Figures 6H,I).

Because *Mir21*^−/−^ mice were more resistant to EAE when compared to WT mice, these data suggested that miR-21 does play a critical role in EAE and therefore, THC+CBD mediated down-regulation of miR-21 may play a role in cannabinoid-mediated attenuation of EAE. However, when we treated *Mir21*^−/−^ mice with THC+CBD, we found that these mice exhibited further reduction in EAE thereby suggesting that additional miRNAs may also be involved in the efficacy of cannabinoids to suppress EAE.

## Discussion

MS is an immune-mediated inflammatory disease of the CNS (41, 42). The precise mechanisms of pathogenesis of MS remain unknown, although environmental as well as genetic components are believed to participate in this demyelinating disease (43). Current treatments for MS often consist of immunosuppressive drugs with many side-effects after prolonged use. Recently, a combination of THC+CBD, extracted from Cannabis plant, named Sativex, has been approved to treat MS in over 28 countries, including Europe and Canada to help improve muscle spasticity (37, 44). Because cannabinoids such as THC and CBD are also potent anti-inflammatory agents (15, 17), the possibility remains that THC+CBD may also suppress neuroinflammation in MS patients. In fact, there is evidence to support this notion in EAE animal models (12, 13), as was also corroborated in the current study. Thus, while cannabinoids may help improve muscle spasticity and attenuate neuroinflammation, the underlying mechanisms remain to be elucidated. In the current study, we investigated the role of miRNA in the attenuation of EAE by a combination of cannabinoids, THC and CBD. Our studies identified several miRs in brain MNCs that targeted inflammatory pathways leading to decreased expression of inflammatory cytokines as well as promoted cell cycle arrest and apoptosis in encephalitogenic T cells in brain. The miRs also promoted Tregs through induction of FoxP3.

We have previously shown that miRNA play a critical role in cannabinoid-mediated suppression of inflammation. In delayed-type hypersensitivity (DTH) model, we noted that THC suppressed Th17 cell differentiation through suppression of miR-21 expression, which induced SMAD7 consequently suppressing Th17 (16). THC also caused downregulation of miR-29b, an IFN-γ inhibitor. THC treatment reversed this miR dysregulation. Additionally, when we transfected primary cells from DTH mice with miR-21 inhibitor or miR-29b mimic, there was an increase in SMAD7 and decrease in IFN-γ expression, respectively. In the current study, we observed downregulation of miR-155 in EAE mice treated with THC+CBD. Recent studies have focused on the participation of miR-155 in EAE. miR-155 mediates inflammatory response through promoting the development of inflammatory Th1 and Th17 cells. Furthermore, miR-155 has been involved in inhibiting the protein suppressor of cytokine signaling 1 (SOCS1) in activated CD4+ T cells (45). Mice lacking miR-155 (mir-155^−/−^) have reduced EAE disease severity accompanied by less CNS inflammation and decreased Th1 and Th17 responses (45). In addition, in the current study, we also noted that cannabinoid treatment led to down-regulation of miR-31, which targeted FoxP3. This is consistent with previous findings that miR-31 targets Foxp3 and it is under expressed in human natural Tregs (46). Also, it has been shown that conditional deletion of miR-31 leads to an increase in peripheral Tregs and reduced severity of EAE (47).

In addition to targeting Tregs and Th17 cells, we also identified some miRs that targeted cell cycle and apoptotic pathways. Studies from our laboratory have shown that THC and CBD when tested individually can trigger apoptosis in immune cells as well as in some cancer cell lines (21, 48–51). However, roles of miRNA in the regulation of cannabinoid-mediated apoptosis in immune cells are not clearly understood, especially with respect to MNCs isolated from the brain during neuroinflammation. In the current study, we noted that THC+CBD treatment led to significant increase in apoptosis in brain MNCs, which also showed decrease in G0/G1 phase of cell cycle and increase in G2M phase. Our results demonstrated that THC+CBD treatment caused downregulation of some miRNAs like miR-122-5p and miR-21a-5p, which may target genes that regulate cell cycle arrest and apoptosis such as *Bcl2L11, CCNG1* and *CDKN2A*, which were found to be upregulated. Moreover, treatment with THC+CBD led to upregulation of miRNAs such as miR-706-5p. It has been reported that miR-706-5p affects the expression of cell division cycle associated 4 gene, *Cdca4*, a gene that is important for cell cycle G1 phase progression specifically through the E2F/retinoblastoma protein pathway (52). Also, miR-706-5p downregulates the activity of *Cacul1*, which is a cell cycle associated protein capable of promoting cell proliferation through the activation of CDK2 at the G1/S phase transition (53).

While THC+CBD treatment led to alterations in many miRNAs, we further focused our studies on miR-21. We observed that *Mir21*^−/−^ mice were more resistant to EAE when compared to WT mice; these data suggested that miR-21 does play a critical role in EAE. These data are consistent with previous studies showing that miR-21 deficiency leads to increased resistance to EAE (54). However, when we treated *Mir21*^−/−^ mice with THC+CBD, we found that these mice exhibited further reduction in EAE thereby suggesting that additional miRNAs may also be involved in the efficacy of cannabinoids to suppress EAE. miR-21 may play a significant role in autoimmune diseases mediated by Th17 cells which is indicated by the fact that its expression is increased in Th17 cells (54). miR-21 promotes Th17 cell differentiation by depleting SMAD-7, a negative regulator of TGF-β signaling (54). miR-21 has also been shown to act as an upstream regulator of IL-10, specifically as a negative regulator of IL-10-producing regulatory B (IL-10^+^ Breg) cells which promote tolerance in autoimmune diseases (55). Thus, miR-21 silencing leads to enhanced differentiation of IL-10^+^ Breg, which attenuate EAE. These findings were also confirmed in another model in which it was shown that specific miR-21 silencing *in vivo* significantly prolonged allograft survival, which was associated with a decrease in Th17 cells and an increase in IL-10^+^ Breg (56). The ability of miR-21 to target IL-10 and Il-17 is also consistent with the observation made in the current study that there was significant down-regulation of miR-21 following cannabinoid treatment and up-regulation of IL-10 and down-regulation of IL-17 in brain-derived CD4+ T cells. Thus, miR-21 may act by downregulating Th17 while inducing IL-10, thereby attenuating EAE.

It is well-established that THC acts through CB1 and CB2 receptors while CBD does not bind to these receptors or binds with very low affinity but *in vivo*, may act through other receptors such as GPR55, TRPV1, 5-HT1a, or PPAR-γ (13, 21–23, 57). Nonetheless, CBD can alter the uptake and breakdown of endocannabinoids thereby indirectly affecting the activation of CB receptors or it can mediate CB1 antagonism (58, 59). Another interesting pathway through which CBD may help suppress neuro-inflammation is through activation of adenosine A_2A_ receptors (60). CBD was shown to attenuate a viral murine model of MS by decreasing the transmigration of blood leukocytes through down-regulation of chemokines and cytokines (61). In this study, use of A_2A_ antagonist blocked some of the anti-inflammatory effects of CBD, thereby demonstrating a key role played by A_2A_ in CBD-mediated suppression of inflammation. In the current study, we used mice deficient in CB1 and CB2 and found that these mice bearing EAE when treated with THC+CBD failed to exhibit EAE amelioration which suggested that THC+CBD treatment was acting through these receptors. However, these mice showed similar levels of clinical disease as the wild-type mice. This may be because they may exhibit some compensatory mechanisms or that the endocannabinoids in the absence of CB1 and CB2 may act on other receptors such as the vanilloid receptors (62). To the best of our knowledge, there are no previous studies on use of such double-knockout mice in EAE. However, use of mice deficient in CB1 or CB2 alone have provided evidence for the involvement of these receptors and endocannabnoids in EAE. For example, mice deficient in CB1 receptor showed a more severe clinical course indicating that endogenous cannabinoids activate CB1 that helps control neuroinflammation and EAE (63). Also, CB2 knockout mice were shown to exhibit exacerbated EAE. However, pharmacological agonism or antagonism of CB2 failed to affect EAE in ABH mice (64). Such studies have raised some concerns about the translational value of some transgenic/gene knockout studies which may depend on susceptibility genetic backgrounds (65). Additionally, the knockout mice may have different microbiota which may influence EAE as shown in our studies in mice with CD44 deletion (66). Thus, clearly, additional studies are necessary on use of CB receptor knock out mice in understanding the role of cannabinoids in EAE.

In the current study, the anti-inflammatory properties of THC+CBD were evident in their ability to decrease the expression of pro- inflammatory cytokines (IL-17A, IL-6, TNF-α, IFNγ, and IL-1β) induced in EAE. Also, Chalah MA et al. found that the pro-inflammatory cytokines IL-6, TNF-α, and IFNγ are related to MS fatigue, which is one of the distinct symptoms that the MS patients are suffering from (67) T-bet is a Th1 cell-specific transcription factor that controls the expression of the hallmark Th1 cytokine, IFN-γ (68). In our study we found that its expression was repressed along with other cytokine transcription factors of IL-17A, IL-6, and TNF-α after treatment with the THC+CBD, which demonstrate the inflammatory suppressive role of the cannabinoids. Increased GATA-3 expression plays an important role in enhancing IL-4 production in differentiated Th2 and inhibiting Th1 differentiation (69). On the other hand, we also found that the cannabinoids increased the expression of the anti-inflammatory cytokines (IL-10 and TGF-β). It has been reported that the deficiency or abnormal expression of IL-10 can increase inflammatory response to microbial challenge but also lead to development of inflammatory bowel diseases (IBDs) and several autoimmune diseases (70, 71). Overall, our data are consistent with the previously reported studies demonstrating that cannabinoids suppress cytokine production and promote Th2 while suppressing Th1 cells (16, 72, 73). Our previous studies have also identified the mechanisms through which cannabinoids such as THC suppress cytokine production. One of the mechanisms include epigenetic modifications in which THC treatment leads to the association of active histone modification signals to Th2 cytokine genes and suppressive modification signals to Th1 cytokine genes, leading to a switch from Th1 to Th2 (74).

While the current study has identified novel miRNA pathways through which cannabinoids suppress neuroinflammation and attenuate EAE, these studies do have some limitations: (1) It is noteworthy that in the current study, while we used a combination THC and CBD, these were pure compounds, whereas in Sativex, the THC+CBD extract from *Cannabis* also includes low levels potentially other minor phytocannabinoids and terpenes, which may enhance the effects of THC+CBD, called the “entourage” effect. Thus, this finding constitutes a limitation in comparing our studies to Sativex. Nonetheless, our studies also demonstrate that pure forms of THC+CBD can also serve as therapeutic modality in the treatment of MS. (2) In the current study, we observed that CBD or THC when administered alone at 10 mg/kg failed to suppress clinical scores in MOG-induced EAE. We found in a previous study that a dose of 20 mg/kg of CBD was necessary to attenuate clinical signs in EAE (15). Our pilot studies and published data showed that CBD dose of 20 mg/kg or higher is necessary to suppress inflammation (15, 23, 57). Thus, in the current study, we used a suboptimal dose of 10 mg/kg each of CBD and THC to test if, when combined, they would work synergistically and suppress neuro-inflammation. Such studies are important because THC is psychoactive, and thus should be used at minimum effective dose, and it is further beneficial if this effect can be augmented by the presence of non-psychoactive CBD, thereby making the combination clinically relevant. There are limited studies on THC and the dose and efficacy may depend on the model of EAE, its use as preventive or treatment measure, the strain/species model used and the like (75). Nonetheless, we found in our current study, using cannabinoids after disease onset, that a single dose of CBD or THC at 10 mg/kg was not effective while a combination of these was highly effective in suppressing clinical symptoms and neuroinflammation in EAE. It should be noted that in a previous study, the authors treated EAE mice with THC (20 mg/kg), CBD (20 mg/kg), or THC+CBD (10 mg/kg each), daily from the day symptoms appeared till the first relapse of the disease and found that the three treatments delayed the onset of symptoms (12). However, only THC+CBD or THC alone were able to attenuate neurological disability while CBD failed (12). The difference between this study and the current study is that we used lower doses of THC or CBD alone (10 mg/kg) and we treated the mice for much shorter duration. The reason for the design of our study was to identify the miRNA which would be induced early on and characterize them. Thus, it is possible that if we had continued treatment with CBD or THC alone for the entire duration of the study, we could have found these to be effective. (3) In a previous study, we noted that CBD at 20 mg/kg could attenuate EAE (15). In the current study, our goal was to try a suboptimal dose of CBD and therefore we used CBD at a dose of 10 mg/kg. The rationale for using suboptimal dose of THC and CBD was to test if they would exert synergistic effect and suppress EAE. It should be noted that in an earlier study, CBD when used at a dose of 5 mg/kg was effective to suppress EAE (76).The reason for the discrepancy between previous study and our study with respect to the dose could be because the authors used only CFA+MOG to trigger EAE (76), while we used PTX+CFA+MOG. PTX is known to enhance EAE induction by promoting robust Th1 and Th17 response (77). This is also evident from the clinical scores because in the previous study, the maximum clinical EAE scores in controls were around 2.5 (76) while with PTX, we get maximum clinical scores around 4.0 in control mice, as seen from our current and past study (15). Also, in our model, we see the earliest signs of EAE around day 10, whereas in the previous study (76), the earliest signs of EAE were seen around day 18. In summary, these observations suggest that lower doses of CBD (5 mg/kg), may be effective in a less severe EAE model that does not use PTX, while higher doses of CBD (20 mg/kg) may be necessary in EAE models induced with PTX, where the disease severity is high. (4) Lastly, the dose of THC used in our study is well within the range used in humans. Based on body surface area normalization guidelines from FDA, 10 mg/kg THC dose in mice converts to 30 mg/m^2^. In humans, THC (Marinol) used as an antiemetic is recommended at a dose of 90 mg/m^2^/day by FDA, which is 3 times higher than what we used in mice.

In conclusion, the current study makes several novel observations that have translational impact in treating patients with MS and other neuro-inflammatory disorders: (1) THC+CBD combination therapy is currently being used to treat MS patients for reducing muscle spasticity. Our studies suggest that such a combination may also suppress neuro-inflammation. Thus, additional clinical studies are necessary to test this finding with varying doses of cannabinoids. It would be beneficial to identify minimum effective dose of THC along with CBD, to prevent undue psychotropic effects of THC. (2) The current study has used cannabinoids to identify several miRNAs that exhibit altered expression in brain infiltrating cells during EAE that suppress inflammation. These miRs target inflammatory cytokines, apoptotic pathways, and promote Tregs by targeting FoxP3. Thus, our studies provide useful information in treating other neurodegenerative diseases driven by chronic neuro-inflammation. (3) The miRs identified serve as novel potential targets for treating MS. Several miR-targeted therapeutics have reached clinical development (78) and thus, downregulation of miRs such as miR-21, may provide a therapeutic pathway to treat MS.

## Data Availability

The data discussed in this publication have been deposited in NCBI's Gene Expression Omnibus (79) and are accessible through GEO Series accession number GSE135317 (https://www.ncbi.nlm.nih.gov/geo/query/acc.cgi?acc=GSE135317).

## Ethics Statement

All animal experiments were ethically performed according to the NIH guidelines and protocols approved by the University of South Carolina Institutional Animal Care and Use Committee.

## Author Contributions

ZA-G, MN, and PN: conceptualization, methodology, and resources. ZA-G: validation and writing—original draft. ZA-G and KM: formal analysis, investigation, and visualization. ZA-G, KM, MN, and PN: writing- review and editing. MN and PN: supervision. ZA-G, MN, and PN: funding acquisition.

### Conflict of Interest Statement

The authors declare that the research was conducted in the absence of any commercial or financial relationships that could be construed as a potential conflict of interest.
